# Phylogeography and population diversity of *Simulium hirtipupa* Lutz (Diptera: Simuliidae) based on mitochondrial COI sequences

**DOI:** 10.1371/journal.pone.0190091

**Published:** 2017-12-27

**Authors:** Vanderly Andrade-Souza, Janisete G. Silva, Neusa Hamada

**Affiliations:** 1 Instituto Nacional de Pesquisas da Amazônia (INPA), Coordenação de Biodiversidade, Laboratório de Citotaxonomia e Insetos Aquáticos, Manaus, Amazonas, Brazil; 2 Universidade Estadual de Santa Cruz, Departamento de Ciências Biológicas, Ilhéus, Bahia, Brazil; National Cheng Kung University, TAIWAN

## Abstract

High morphological homogeneity and cryptic speciation may cause the diversity within Simuliidae to be underestimated. Recent molecular studies on population genetics and phylogeography have contributed to reveal which factors influenced the diversity within this group. This study aimed at examining the genetic diversity of *Simulium hirtipupa* Lutz, 1910 in populations from the biomes Caatinga, Cerrado, and Atlantic Forest. In this study, we carried out phylogeographic and population genetic analyses using a fragment of the mitochondrial gene COI. The 19 populations studied were clustered into seven groups, most of which are associated with geography indicating certain genetic structure. The northern region of the state of Minas Gerais is most likely the center of origin of this species. The average intergroup genetic distance was 3.7%, indicating the presence of cryptic species. The species tree as well as the haplotype network recovered all groups forming two major groups: the first comprises groups Gr-Bahia (in which the São Francisco river has not acted as geographical barrier), Gr-Pernambuco, and Gr-Mato Grosso do Sul. The second included groups comprising populations of the states of Goiás, Tocantins, Minas Gerais, Bahia, São Paulo, and Espírito Santo. The mismatch distribution for groups was consistent with the model of demographic expansion, except for the Gr-Central-East_1 group. The diversification in this group occurred about 1.19 Mya during the Pleistocene, influenced by paleoclimatic oscillations during the Quaternary glacial cycles.

## Introduction

The family Simuliidae has a wide geographic distribution occurring in all continents except Antarctica. Over 25 species are of medical and veterinary importance since black flies are etiological agents of human onchocerciasis. As well, high biting activities of females can reduce animal productivity of birds and mammals. Moreover, their immature stages play an important role in nutrient turnover in streams. Due to the extensive cryptic speciation and high morphological homogeneity observed in the Simuliidae, much of the diversity within this group may be underestimated [1–6].

Morphological and cytotaxonomic studies that have been historically used to assess the biodiversity of this group, and recently molecular markers have been incorporated into studies of systematics, phylogeny, population genetics, and phylogeography [2]. Nonetheless, information on the genetic diversity of this group is still largely unknown due to the high morphological similarity among the taxa [2,7–11]. Recognition of multiple lineages within taxa is paramount for the understanding of evolutionary processes. However, the recognition of these lineages within morphologically conserved taxa can be very challenging due to their low genetic differentiation [12].

DNA barcode studies using a small portion of the gene cytochrome c oxidase subunit I (COI) for species identification [13] have proven successful in many animal groups. Since there are many difficulties with morphological and chromosomal identifications coupled with a shortage of taxonomists and cytogeneticists working on this group, simuliids are an excellent group to utilize DNA-based identification system for species-level identification [6]. However, studies on Neotropical simuliids using this molecular tool are still scarce [14].

In simuliids, the DNA barcode approach has been used in studies focusing on phylogeny [15]; population genetics [16–18], and phylogeography [10,19,20], as well as revealing cryptic diversity [5–7,14,21]. It has also been used in conjunction with other mitochondrial and nuclear markers [12,22].

Phylogeographic studies that combine genetic and geographic data, have allowed inferences to be made on vicariance and dispersal processes that drive animal and plant species distributions [23]. Aquatic insects are still under-represented in phylogeographic studies, however, they are ideal for this kind of research. They vary widely in dispersal abilities and many share similar geographic distributions, which can allow the confirmation of inferred vicariance events in several species [24]. Within simuliids only a few phylogeographic studies have been carried out [e.g. 10,20], and none have included populations from the Neotropical region.

*Simulium hirtipupa* Lutz, 1910 is restricted to Brazil where it is present in four of the five major geographic regions, it is not present in the North region [25,26]. Recently a new species previously identified as a northern population within this nominal species was described as *Simulium criniferum* [26]. It occurs in the Brazilian biomes of Caatinga, Cerrado, and the Atlantic Forest, which correspond to the occurrence area of Seasonally Dry Tropical Forests (SDTFs), one of the most endangered ecosystems due to heavy deforestation [27]. In the Neotropical region there is a lack of phylogeographic knowledge on simuliids. The characteristics of *S*. *hirtipupa* mentioned above may contribute to the understanding of which environmental and geological factors have most likely influenced the diversification and dispersal of this group in the Neotropical region.

Cryptic differentiation is common to many Simuliidae species [28]. Accordingly, the genus *Simulium* comprises several species complexes [1,6,14]. Cryptic taxa can however display key differences that are important for ecological and epidemiological reasons, since the larvae of some species are important bioindicators and adults of some species are vectors of diseases, respectively [2,6,28–30]. Therefore, accurate identification at the species level is paramount for biomonitoring and control programmes [6]. So far, the feeding habits of females of *S*. *hirtipupa* are unknown, although they are thought to be zoophilic [31]. The recently described *S*. *criniferum* is the closest relative to *S*. *hirtipupa* and the females of the former are anthropophilic [26]. These results suggest that the genetic patterns of *S*. *hirtipupa* might be both more complex than previously hypothesized and could also reflect interactions between cryptic differentiation and dispersal. Thus, in order to examine the genetic diversity and to elucidate the diversification process of this nominal species of Brazilian simuliid, we utilized a fragment of the mitochondrial gene COI and present here a large-scale phylogeographic and population genetic analyses of *S*. *hirtipupa*. Specifically, the objectives of this study are threefold: (i) examine the population structure and genetic diversity of *S*. *hirtipupa* across a large part of its distribution), (ii) infer the time of divergence and demographic history, and (iii) verify the occurrence of cryptic taxa.

## Materials and methods

### Ethics statement

Black fly collections were made on public or owner-permitted private lands. A permit to collect zoological specimens was provided by SISBIO (permanent permit number 10873–1 to NH). No collections involved endangered or protected species.

### Data collection, extraction, amplification, and sequencing

**Fig 1:**
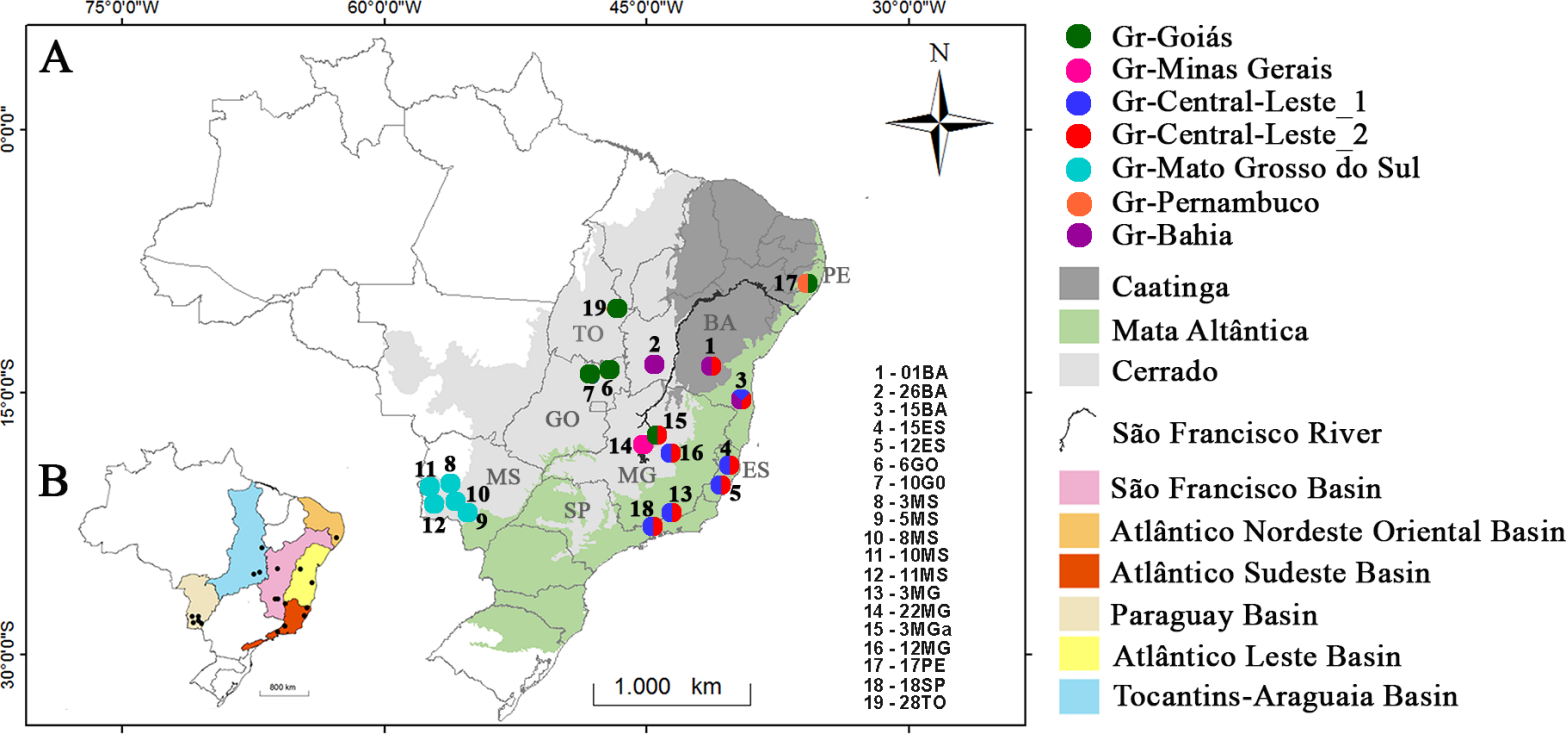
Geographic distribution and sampling sites of the populations of *Simulium hirtipupa* included in this study. A) Map of Brazil with sampling sites and the respective biomes. B) Map of Brazil showing the sampling sites in their respective river basins (Resolution n° 32, from Conselho Nacional de Recursos Hídricos, of October 15, 2003). For details on localities and population codes see Table 1. The Brazilian states are: BA, Bahia; ES, Espírito Santo; GO, Goiás; MS, Mato Grosso do Sul; MG, Minas Gerais; PE, Pernambuco; SP, São Paulo; TO, Tocantins.

Sequences of 19 populations of *S*. *hirtipupa* (Table 1, Fig 1) from eight states in Brazil encompassing the biomes of Caatinga, Cerrado, and the Atlantic Forest were analyzed. These populations were collected in six river basins located on both banks of the São Francisco river, the largest entirely Brazilian river and one of the longest rivers in South America. The São Francisco river is flanked by mountain ranges such as the Cadeia do Espinhaço and Serra Geral de Goiás [32].

**Table 1:** Collection data and information on *Simulium hirtipupa* populations sequenced in this study. N—number of sequences.

Locality	Code	Collection date	N	Elevation (m)
**Bahia—BA**				
Ibicoara—Balneário do Oton 41°15’7.34"W; 13°26’54”S	**1BA**	23/07/2006	8	902
Correntina—Rio Correntina 44°36’08.0”W; 13°19’59.6”S	**26BA**	07/08/2010	15	514
Camacan—Rio Panelão 39°32’01.8”W; 15°24’24.9”S	**15BA**	02/08/2010	15	179
**Espírito Santo—ES**				
Linhares—Cachoeira de Angelis 40°14’24.9”W; 19°7’31.1”S	**15ES**	08/11/2011	13	36
Domingos Martins—Cachoeira do Galo 40°38’58.61”W; 20°17’21.02”S	**12ES**	16/02/2012	13	574
**Goiás—GO**				
Teresina de Goiás—Cachoeira do poço Encantado 47°15’38.5”W; 13°52’30.6”S	**6GO**	02/06/2007 24/06/2009	27	798
Colinas do Sul—Cachoeira da Igrejinha 48°5’10.2”W; 14°8’56.1”S	**10GO**	03/06/2007	15	516
**Mato Grosso do Sul—MS**				
Bonito—Rio Formoso 56°26’13.3”W; 21°10’02.4”S	**3MS**	14/03/2012	14	268
Bonito—Rio Chapenha 56°33’15.73”W; 20°49’53.09”S	**5MS**	15/03/2012	12	313
Bonito—Balneário Ilha Bonita 56°23’59.8”W; 21°08’41.4”S	**8MS**	16/03/2012	14	252
Bonito—Rio Mimoso 56°30’38.7”W; 20°59’57.4”S	**10MS**	16/03/2012	14	382
Bonito—Rio Betione 56°39’01.9”W; 20°34’56.9”S	**11MS**	17/03/2012	15	260
**Minas Gerais—MG**				
Juiz de Fora—Jacutinga 43°29’46.2”W; 21°50’28.4”S	**3MG**	19/06/2010	16	689
Lassance—Cachoeira Tapera 44°58'34.3"W; 17°50'02.3"S	**22MG**	07/06/2013	15	551
Lassance—Ribeirão do Nozão 44°32'12.6"W; 17°47'46.4"S	**3MGa**	31/05/2014	5	511
Serro—Cachoeira do Beijo 43°28'04.5"W; 18°31'31.0"S	**12MG**	07/06/2014	13	953
**Pernambuco—PE**				
São Benedito do Sul—Cachoeira do Peri-Peri 35°54’3.69”W; 08°47’3.85”S	**17PE**	31/07/2006	29	470
**São Paulo—SP**				
São José do Barreiro—Córrego Pau d’Alho 44°36’56”W; 22°38’10”S	**18SP**	13/05/2004	15	498
**Tocantins—TO**				
Jalapão—Rio Novo, Cachoeira da Velha 46°52’50.7”W; 10°16’11.8”S	**28TO**	21/08/2002	15	312

Collectors: Hamada, N.; Pepinelli, M. (populations 1BA, 17PE, and 18SP); Hamada, N. (populations 26BA and 15BA); Nascimento, J.M. (population 12ES); Hamada, N., Nascimento, J.M. (populations22MG, 3MGa, and 12MG); Cruz, P.V., Del Carro, K. B (population 15ES); Hamada, N., Zampiva, N.K. (populations 3MS, 5MS, 8MS, 10MS, and 11MS); Hamada, N., Pereira, E.S. (populations 6GO and 10G); Hamada, N., Oliveira, V.C. (population 3MG); Hamada, N., Gomes, N.S., Silva, J.O. (population 6GO); Hamada, N., Kikuchi, R., Ronchi-Teles, B. (population 28TO).

Larvae and pupae were hand collected directly from the available substrate, preserved in absolute ethanol and kept refrigerated at -20°C at the Laboratory of Citotaxonomia de Insetos Aquáticos, Coordenação de Biodiversidade, Instituto Nacional de Pesquisas da Amazônia, where the material was identified.

From 8 to 29 specimens per population (Table 1) were used for DNA extraction using the DNeasy Blood & Tissue kit (Qiagen) per the manufacturer’s instructions. DNA was extracted using the nondestructive method thus allowing the exuviae to be kept in ethanol as vouchers, which are deposited in the above mentioned laboratory.

PCRs were performed using the Swift™ Maxi Thermal Cycler (ESCO Technologies Inc., USA) and TC-512 Techne Thermal Cycler (Techne Inc., Staffordshire, UK), 25 μl reaction volumes and 1–3 μl of DNA template. Each reaction had a final concentration of 0.5X buffer, 3mM MgCl_2_, 0.25mM of each dNTP, 0.96μM of each primer, and 1.5U of *Taq* polymerase (Promega). The COI gene was amplified from all DNA samples using the Folmer et al. [33] forward and reverse primers. Cycling conditions were 3 min at 94°C followed by 36 cycles of 94°C (1 min) / 45°C (1.5 min) / 72°C (1.5 min) and an extension of 5 min at 72°C. PCR products stained with GelRed^TM^ visualized on 1% agarose gels. Samples were then purified using ExoI and SAP (Fermentas@) at 37°C (30 min) and 80°C (15 min). DNA sequences were generated using an ABI 3730 DNA Analyzer at the Centro de Estudos do Genoma Humano at the Universidade de São Paulo (CEGH-USP).

All DNA sequence trace files were edited using BioEdit v. 5.0.6 [34]. The best model of sequence evolution for distance was chosen using Modeltest 3.06 [35]. The best-fit model inferred for COI was the GTR+I+G with Gamma shape 0.647. The K_2_P model, with Gamma shape 0.603, was used to calculate pairwise distances using MEGA 6 and Arlequin 3.5.

### Population structure and genetic diversity

The Bayesian population mixture analysis was implemented in BAPS v. 4.13 5.3.1 (*Bayesian Analysis of Population Structure*) [36] to identify the population genetic grouping. First a mixture analysis was carried out and the results were used in the admixture analysis, the number of groups (k) was set from 2 to 10.

Basic statistics of mtDNA diversity, including nucleotide and haplotype diversity, analysis of molecular variance (AMOVA), pairwise Fst estimates, as well as Tajima’s *D* [37] and Fu’s *Fs* [38] neutrality tests were calculated with Arlequin 3.5 [39] and compared across all geographic groups. These neutrality tests were used to test population equilibrium and are expected to yield high negative values for expanding populations [40].

Demographic history was inferred based on estimated and observed mismatch distributions and calculated Sum of Squared Deviations (SSD) and Harpending’s raggedness index (rg) also in Arlequin 3.5 [39]. The correlation between genetic and geographic distances among the populations was also evaluated using Mantel Tests [41] with 10,000 randomizations. Intra- and inter-population genetic distances as well as intra- and inter-group distances were calculated using MEGA6 [42] following the model described above.

A haplotype network was constructed using median joining (MJ) method Network 4.6.0.0 (Fluxus Technology Ltd, downloaded from http://www.fluxustechnology.com/sharenet.htm) to infer the relationships among haplotypes and their geographical distribution.

### Time of divergence and demographic history

The Bayesian species tree and the time of divergence among populations were estimated using BEAST 1.8.2 6 (Bayesian Evolutionary Analysis Sampling Trees) [43]. In this analysis, a molecular clock with a standard insect mitochondrial divergence rate (2.3% per million years) was implemented [44] under the relaxed clock (uncorrelated lognormal) mode for rate variation among lineages. Five replicate runs were performed with 300 million generations. Tracer v1.6 [45] was used to verify convergence (ESS—Effective Sample Size > 200). The resulting trees were combined in the LogCombiner v1.8.2 [43] after a burn-in of 10%. The maximum clade credibility tree was accessed using TreeAnnotator v1.8.2 [43]. The tree was visualized and edited using FigTree v.1.4.1 (http://tree.bio.ed.ac.uk/software/figtree/).

A continuous Bayesian phylogeography approach under a lognormal relaxed random walk (RRW model) diffusion model across continuous space was applied using BEAST v1.8.2 to infer the historical dispersal routes. The maximum clade credibility tree was used to generate a visual representation of the historical movements in Google Earth v7.1.5 (Google Inc.) using the Continuous Tree module in SPREAD v1.0.662 [46].

## Results

### Population structure and genetic diversity

Our sequence data comprised a 651bp fragment of COI mtDNA from 284 specimens of *Simulium hirtipupa* from 19 populations in Brazil. Of the 121 base substitutions identified, a total of 98 were transitions and 23 were transversions. The sequenced region contained 153 polymorphic sites defining 148 haplotypes, which ranged from 5 to 22 per population (Table 2). Sequences were deposited at GenBank under accession numbers MF043593-MF043740 (S1 Table).

**Table 2:** Summary of genetic diversity measures and neutrality tests for the complete set of sequences and for the seven populations of *Simulium hirtipupa*. Hp, haplotype Number; NS, polymorphic sites number; π±SD and H±SD, nucleotide and haplotype diversities, with respective standard deviations.

Population	HP	NS	π±SD	H±SD	Tajima's D	Fu's FS
1BA	7	31	0.023 ± 0.013	0.964 ± 0.077	-	-
26BA	13	32	0.013 ± 0.007	0.886 ± 0.069	-0.8188	-8.8064*
15BA	9	33	0.015 ± 0.008	0.981 ± 0.031	-0.336	-7.8267*
15ES	10	10	0.007 ± 0.004	0.897 ± 0.054	1.7586	-9.6671*
12ES	9	17	0.007 ± 0.004	0.923 ± 0.069	-0.764	-10.0929*
6GO	7	7	0.002 ± 0.001	0.664 ± 0.088	-1.3006	-30.2370*
10GO	5	5	0.002 ± 0.001	0.638 ± 0.129	-1.1587	-27.7766*
3MS	13	18	0.007 ± 0.004	0.989 ± 0.031	-1.0066	-11.7892*
5MS	11	15	0.006 ± 0.003	0.985 ± 0.040	-1.1896	-10.1399*
8MS	9	15	0.005 ± 0.003	0.978 ± 0.035	-1.1422	-13.2548*
10MS	12	13	0.005 ± 0.003	0.952 ± 0.040	-0.8224	-15.8540*
11MS	11	15	0.005 ± 0.003	0.912 ± 0.059	-1.1517	-13.6093*
3MG	6	15	0.008 ± 0.005	0.850 ± 0.054	0.6322	-12.9992*
22MG	11	19	0.006 ± 0.004	0.933 ± 0.054	-1.2735	-13.7696*
3MGa	4	21	0.028 ± 0.017	0.900 ± 0.161	-	-
12MG	11	30	0.007 ± 0.004	0.962 ± 0.050	1.1423*	0.3002*
17PE	23	43	0.008 ± 0.004	0.959 ± 0.027	-2.0129*	-25.5566*
18SP	6	13	0.009 ± 0.005	0.848 ± 0.054	1.7628	-10.9265*
28TO	14	18	0.005 ± 0.003	0.990 ± 0.028	-1.8810*	-16.4207*
Gr-Goiás	23	27	0.003 ± 0.002	0.841 ± 0.034	-2.2510*	-27.5642*
Gr-Minas Gerais	11	19	0.006 ± 0.004	0.933 ± 0.054	-1.2735	-13.7696*
Gr-Central-East_1	6	7	0.003 ± 0.002	0.658 ± 0.096	0.4307	-26.8982*
Gr-Central-East_2	29	32	0.006 ± 0.003	0.912 ± 0.021	-1.3239	-25.9566*
Gr-Mato Grosso do Sul	36	32	0.006 ± 0.003	0.971 ± 0.008	-1.5324*	-26.1020*
Gr-Pernambuco	22	24	0.005 ± 0.003	0.956 ± 0.029	-1.5872*	-26.1058*
Gr-Bahia	19	41	0.015 ± 0.008	0.990 ± 0.018	-0.5314	-13.6324*
Total	148	108	0.032 ± 0.016	0.984 ± 0.003	0.3366	-23.6042*

*p < 0.05.—Index not calculated due to low sample number

The BAPS analysis divided the populations into seven groups (Table 3, Fig 1). Only the groups Gr-Minas Gerais, Gr-Mato Grosso do Sul, and Gr-Pernambuco, which are located in distinct river basins, comprised haplotypes exclusive to one biome (Fig 1B). This finding contrasts with the other groups: Gr-Goiás (populations 6GO, 10GO - 28TO - 3MGa - 17PE); Gr-Minas Gerais (populations 22MG); Gr-Central-East_1 (populations 15BA, 12ES, 15ES, 3MG, 12MG and 18SP); Gr-Central-East_1 (1BA, 15BA, 12ES, 15ES, 3MG, 3MGa, 12MG and 18SP), which comprise haplotypes present in more than one biome. The groups and the populations also showed differences in the genetic diversity indices.

**Table 3:** Population clusters of *Simulium hirtipupa* found by BAPS. Population codes are according to Table 1.

Groups	Populations†
1. Gr-Goiás	6GO - 10GO - 28TO - 3MGa - 17PE
2. Gr-Minas Gerais	22MG
3. Gr-Central-East_1	15BA - 12ES - 15ES - 3MG - 12MG - 18SP
4. Gr-Central-East_2	1BA - 15BA - 12ES - 15ES - 3MG - 3MGa - 12MG - 18SP
5. Gr-Mato Grosso do Sul	3MS - 5MS - 8MS - 10MS - 11MS
6. Gr-Pernambuco	17PE
7. Gr-Bahia	1BA - 15BA - 26BA

† The underlined codes correspond to populations with haplotypes that occurred in more than one group.

Haplotype and nucleotide diversities were used as measures of genetic diversity in *S*. *hirtipupa* (Table 2). General haplotype diversity (H) was 0.984 ranging from 0.638 in the population 10GO to 0.99 in the population 28TO, from the states of Goiás and Tocantins, respectively. In most populations, this index ranged from moderate to high (greater than 0.9). In the group analysis, haplotype diversity ranged from 0.658 in the group Gr-Central-East_1 to 0.99 in the group Gr-Bahia (Table 2). The nucleotide diversity for all sequences was high, but for populations in general was low, ranging from 0.002 in populations 10GS and 06GO to 0.028 in population 3MGa. Within groups the variation ranged from 0.003 in the groups Gr-Goiás and Gr-Central-East_1 to 0.015 in the group Gr-Bahia.

**Fig 2:**
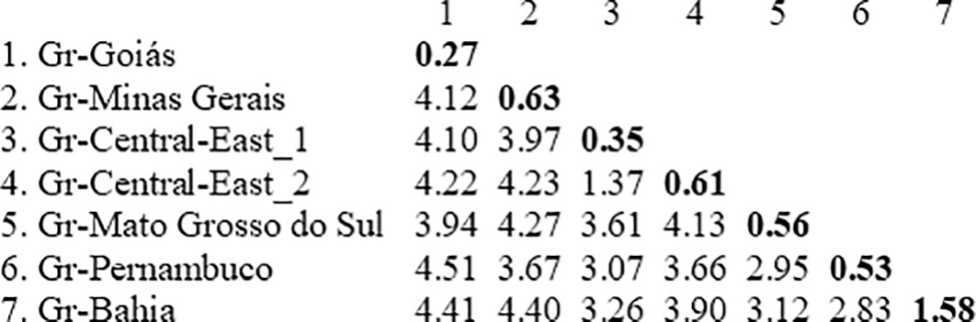
Estimate of genetic distance of *Simulium hirtipupa*. Intragroup (diagonal and bold) and intergroup (below the diagonal) genetic distances (%) based on sequencing of a fragment of the mitochondrial COI gene. Analyses were conducted using the K_2_P model.

The average intrapopulation genetic distance was 0.88% ranging from 0.16% in populations 10GS and 06GO from the state of Goiás to 2.76% in the population 3MGa from the state of Minas Gerais. The average interpopulation distance was 3.3%, ranging from 0.2% between populations 10GS and 06GO from the state of Goiás to 4.6% between populations 28TO and 26BA from the states of Tocantins and Bahia, respectively (S1 Fig). This intragroup index ranged from 0.27% in Gr-Goiás to 1.58% in Gr-Bahia and among groups that average was 3.7%, ranging from 1.37% to 4.5% between the groups Gr-Central-East_1 and Gr-Central-East_2 and between the groups Gr-Goiás and Gr-Pernambuco, respectively (Fig 2).

The Mantel test results indicated a weak correlation between genetic and geographic distance (*r* = 0.613, P = 0.00). For instance, the genetic distance between the groups Gr-Pernambuco and Gr-Goiás (4.51%) was higher than that between the groups Gr-Pernambuco and Gr-Mato Grosso do Sul (2.95%).

**Fig 3:**
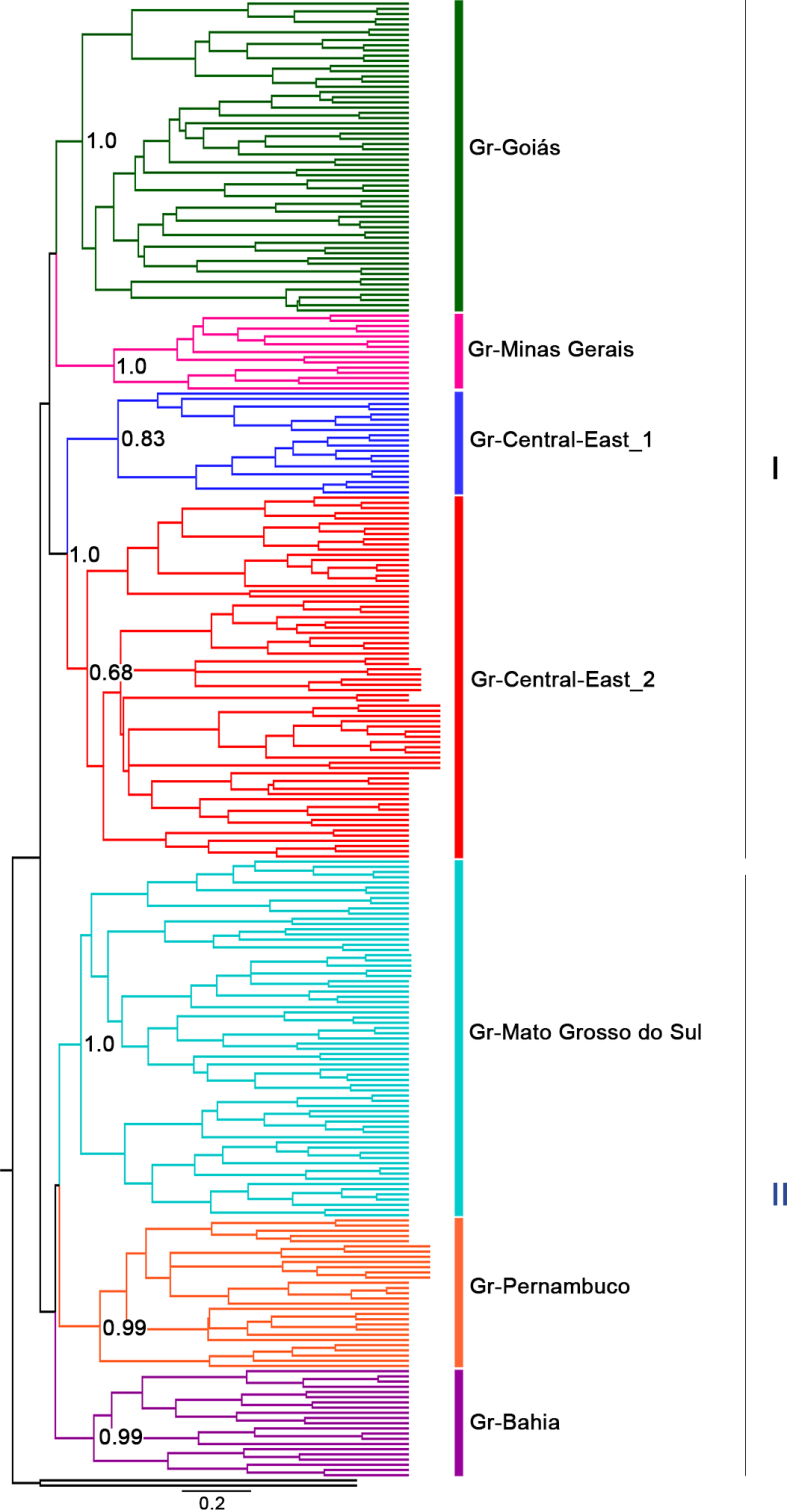
Bayesian inference tree and posterior probabilities based on sequencing of a fragment of the mitochondrial COI gene. Numbers below each node indicate Bayesian posterior probabilities. *Simulium guianense* and *Simulium rubrithorax* were used as outgroups.

The species tree recovered the same seven groups obtained in the BAPS analysis, which formed two major groups: I) Gr-Goiás, Gr-Minas Gerais, Gr-Central-East_1, and Gr-Central-East_2, and II) Gr-Mato Grosso do Sul, Gr-Pernambuco, and Gr-Bahia. Most groups had support values higher than 0.95, except for the group Gr-Central-East_2, whose support value was 0.68. However, the group Gr-Central-East_2 clustered together with the group Gr-Central-East_1 with high support value, both of which comprise haplotypes from shared populations (Fig 3). On the other hand, support values were relatively low for some groups such as groups Gr-Goiás and Gr-Minas Gerais (0.39) and Gr-Mato Grosso do Sul and Gr-Pernambuco (0.27).

**Fig 4:**
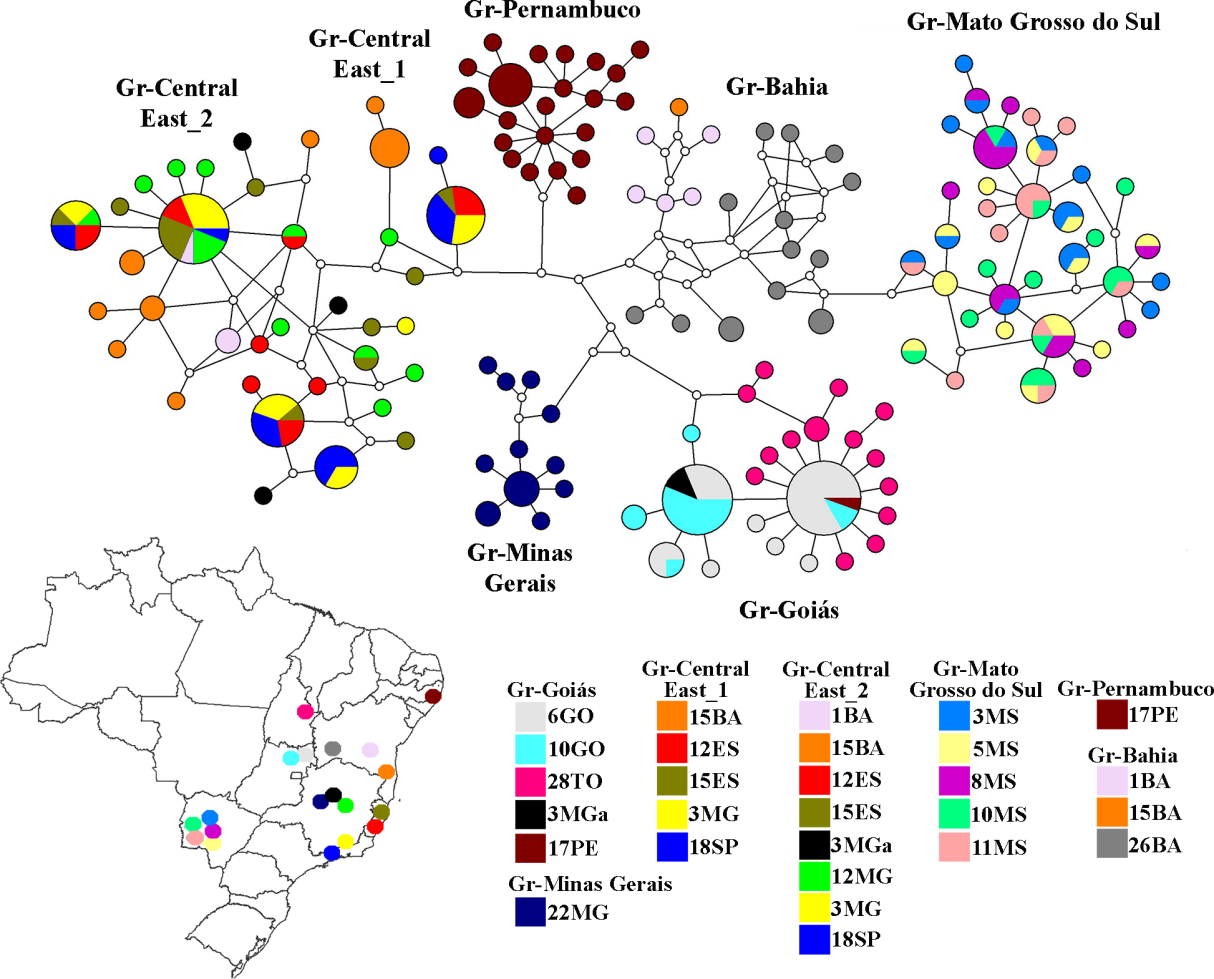
Unrooted haplotype network of *Simulium hirtipupa* based on 651 bp of a fragment of the mitochondrial COI. Each circle in the haplotype network corresponds to one haplotype, and its size is proportional to its frequency among the samples. Colors of the circles correspond to sampling locations. Empty circles are median vector that represent intermediate haplotypes that were not sampled or extinct. On the map the circles are labeled according to the geographic origin of the haplotypes.

The haplotype network (Fig 4) exhibited a certain level of genetic structure and concordance with the species tree. There was haplotype sharing among populations and some of them comprised haplotypes from distinct groups. Only populations 15BA, 26BA, and 22MG had exclusive haplotypes. Haplotype sharing among populations in group Gr-Central-East_2 suggests the occurrence of gene flow, which may still be taking place as recent dispersal routes have been inferred from the spatial and temporal analysis (see below). Ongoing gene flow was also detected among populations within the group Gr-Mato Grosso do Sul, which are geographically closer (Fig 1). The group Gr-Bahia showed the highest number of mutational steps among haplotypes, as well as a larger number of either unsampled or extinct haplotypes.

The hierarchical analysis revealed significant genetic variation between groups and within populations indicating high level of genetic structuring and recent restricted gene flow between the groups except between groups Gr-Central-East_1 and Gr-Central-East_2, which comprise the same populations.

Genetic differences among groups explained 83.12% of the total variation (*F*_*CT*_ = 0.83, p = 0.00) followed by variation within populations of 13.66% (*F*_*ST*_ = 0.86). Only 3.22% of the variation was attributed to variation among populations within groups (*F*_*SC*_ = 0.19) (Table 4). These results were corroborated with the pairwise *Fst* (P = 0.05) values. They showed significant genetic differentiation among most populations (88%) with pairwise values ranging between -0.05 and 0.93 (S2 Fig). Exception to this differentiation amongst populations was found between the populations within the group Gr-Mato Grosso do Sul, between populations 1BA and 3MGa; 3MG and 12MG; 18SP, 3MG and 12ES; 3MG, 12MG and 12ES; and 15ES compared to populations 15BA, 12ES, 3MG, 12MG e 3MGa, which did not show any differentiation. We found differentiation among all groups with *Fst* values ranging from 0.58 to 0.96.

**Table 4:** Partitioning of DNA variance as revealed by analysis of molecular variance (AMOVA) based on COI sequences for *Simulium hirtipupa* sampled in Brazil found by BAPS.

Source of variation	Percentage of variation	Fixation Index
Among groups	83.12	F_CT_ = 0.8311*
Among population within groups	3.22	F_SC_ = 0.1908*
Within populations	13.66	F_ST_ = 0.8634*

*P < 0.05

The Tajima’s *D* value for all individuals was positive and non significant, as well as for most populations and groups. The exceptions, where the Tajima’s *D* values were negative and significant for populations, were 12MG, 28TO, and 17PE and for groups Gr-Goiás, Gr-Mato Grosso do Sul, and Gr-Pernambuco (Table 2). Fu's *Fs* test is more sensitive for detecting population expansion [38]. Fu's *Fs* test values were negative and significant for all individuals, populations and groups analyzed, thus indicating that populations experienced demographic expansion (Table 2).

### Time of divergence and demographic history

**Fig 5:**
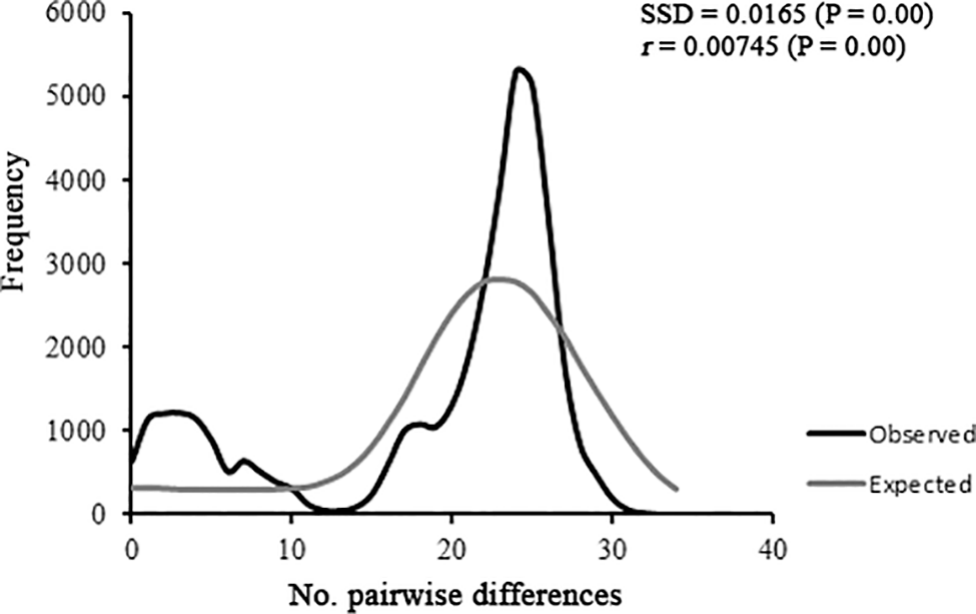
Graph of the mismatch distribution of all populations of *Simulium hirtipupa*. The simulated distribution does not fit the model of population expansion. The lines represent the observed (black line) and expected (grey line) frequency of pairwise differences under the sudden population expansion model.

The mismatch distribution for all populations was multimodal (Fig 5). Conversely, the mismatch distribution for groups was unimodal, which was consistent with the model of demographic expansion (S3 Fig). The only exception was the group Gr-Central-East_1, which exhibited a multimodal mismatch distribution pattern. This indicates that this group comprises populations that are genetically distinct, although the mismatch distribution did not differ significantly from the expected distribution of a sudden population expansion (SSD = 0.08, p = 0.07 e r = 0.16, p = 0.16).

**Fig 6:**
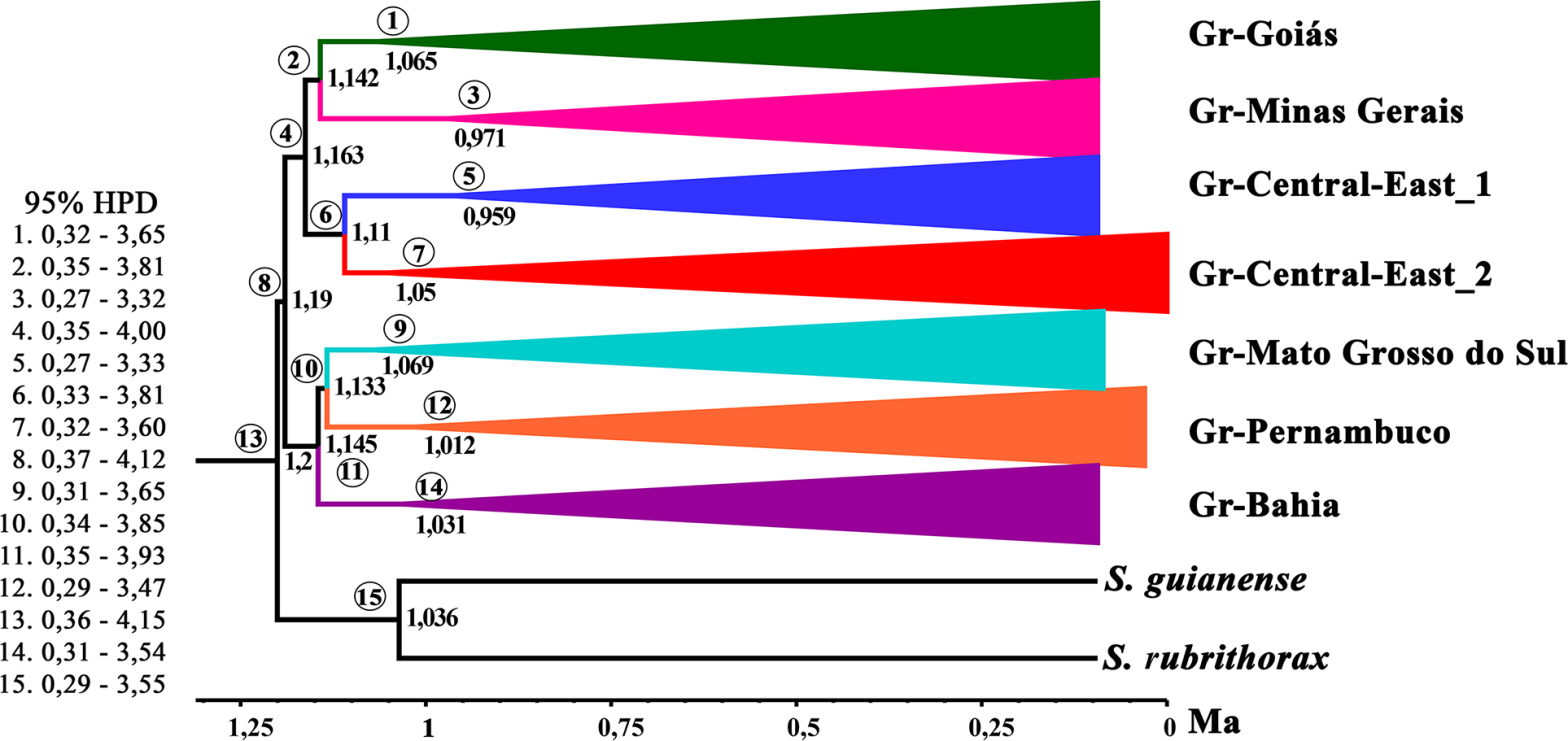
Divergence time estimate for *Simulium hirtipupa* based on a relaxed molecular clock (uncorrelated lognormal). Numbers in each node indicate estimated ages in millions of years ago (Mya). *Simulium guianense* and *Simulium rubrithorax* were used as outgroups. In the right are 95% HPD interval of date estimates.

**Fig 7:**
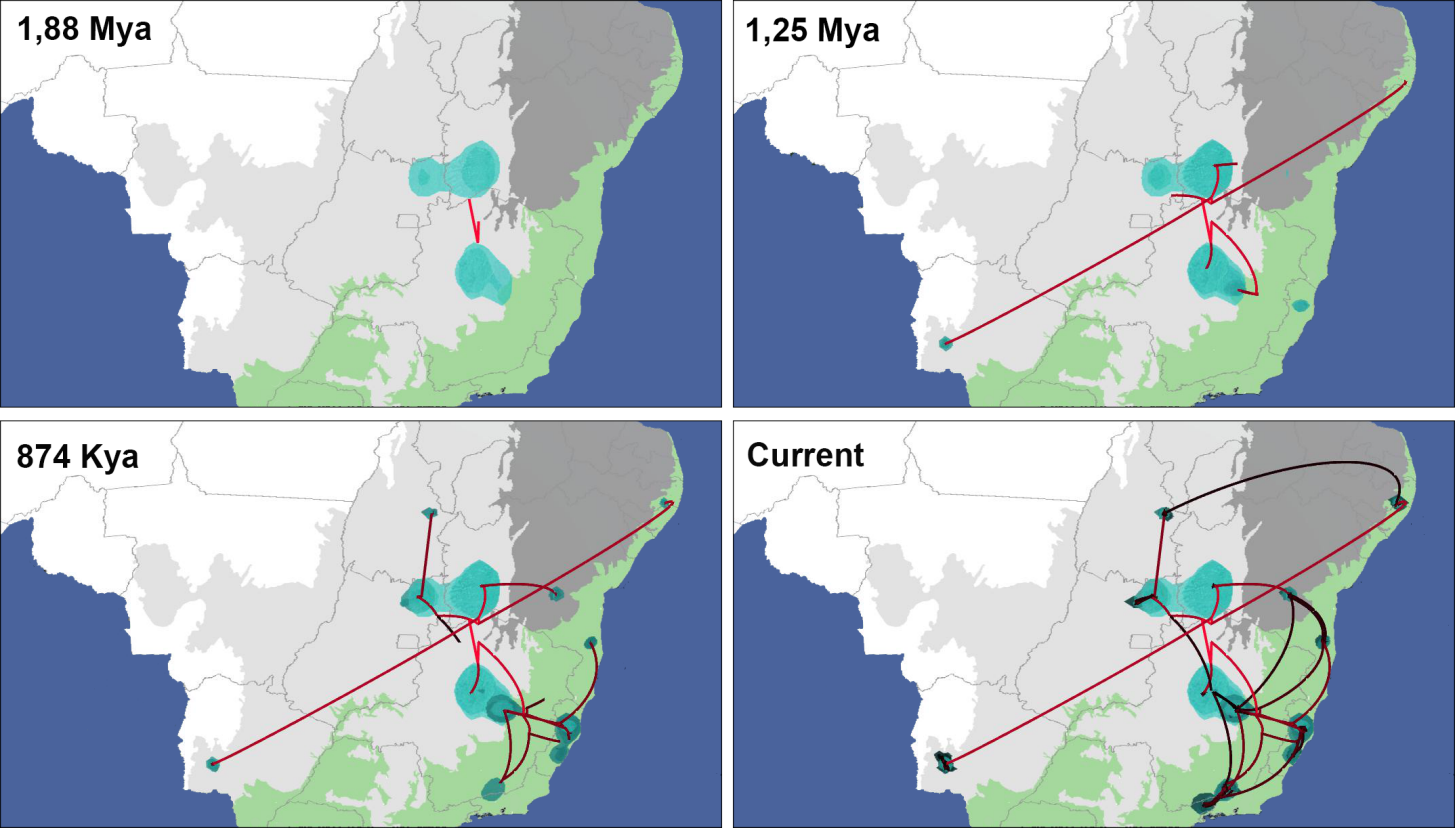
Bayesian Spatiotemporal model of *Simulium hirtipupa* at four time slices. Reconstructions are based on the maximum clade credibility tree estimated with a time-heterogeneous relaxed random walk approach (RRW). Shading represents 80% HPD uncertainty in the location of ancestral branches with lighter and darker shades representing older and younger diffusion events, respectively. The map was visualized from the.kml file provided by SPREAD software generated using Google Earth (http://earth.google.com.

Our results indicate that genetic differentiation in this group occurred about 1.19 Mya (Fig 6). Moreover, our findings also suggest that the center of origin of *S*. *hirtipupa* is in the northern region of the state of Minas Gerais close to the municipality of Lassance, where the type locality is situated and also where populations 22MG and 3MGa were collected. Our data also indicate that dispersal occurred from northern Minas Gerais to other regions of Brazil around 1.88 Mya (Fig 7).

## Discussion

Our results revealed two major groups within *Simulium hirtipupa*: one group (I) comprising populations from the states of Pernambuco, Mato Grosso do Sul, and Bahia, and a second group (II) clustering populations from the states of Goiás, Tocantins, Minas Gerais, Espírito Santo, São Paulo, and also Bahia. Our data also indicate that genetic differentiation in *S*. *hirtipupa* occurred about 1.19 Mya during the Pleistocene when the two major groups might have been formed, the first around 1.16 Mya and the second around 1.14 Mya. The diversification of simuliids has occurred more recently such as in *Simulium angulistylum* (930,000 years ago) [40], *Simulium tani* (500,000 years ago) [10], *Simulium siamense* (120,000 years ago) [19] and *Simulium aureohirtum* (18,000 years ago) [20].

Genetic distance values revealed in this study indicate the presence of cryptic species within *S*. *hirtipupa*, since 65% of the distance values between populations were higher than 3.5%, which is the same value found among most groups. Previous studies have indicated the presence of cryptic species within Simuliidae with genetic distances of 4.5–6.5% [6] and 3.2–3.7% [14]. Moreover, those studies on Simuliidae have revealed maximum intraspecific genetic distances of 3.84% [6] and 9.27% [5], and interspecific genetic distances from 2.83 to 28.6% [5,6,14,47]. In contrast, closely related species showed genetic distance values lower than 0.34% [5].

Our results indicate the likely presence of cryptic species within *S*. *hirtipupa*. The genetic distance and the presence of exclusive haplotypes in group Gr-Minas Gerais provide robust support for considering this lineage as a cryptic species. Since most groups comprise specimens from different populations, further studies are necessary in order to point out which other populations may represent cryptic species. For instance, a chromosome analysis of populations from the states of Bahia and Goiás showed that they differed from each other in one fixed inversion in three chromosomes, therefore indicating that *S*. *hirtipupa*, is a complex with at least two cytoforms [26].

Populations 22MG (Gr-Minas Gerais) and 3MGa showed a strong molecular divergence with average genetic distance of 4.2% (S1 Fig), which indicates that population 22MG can be regarded as a cryptic species. These populations from the central region of the state of Minas Gerais are 46 km apart and separated by the Rio das Velhas river, a tributary of the São Francisco river with an average width of 38.3m [48].

Population 22MG is relatively more recent than the other populations. According to the divergence time estimate (Fig 6), population 22MG diverged 0.971 Mya, which makes this group the second most recent following Gr-Central-East_1 group (0.959 Mya), which was the most recent. Considered together, both the lack of haplotype sharing and the greater genetic distance of this population in relation to the other populations, strongly indicate absence of gene flow. This absence of gene flow might have been due to environmental conditions. On one occasion we noticed a significant reduction in the river’s water flow during sampling, which might have forced the females to oviposit in or near their natal habitat instead of dispersing. In simuliids, site fidelity may play an important role in local adaptation, which may lead to isolation, population differentiation, and, ultimately to speciation [17,28].

The genetic diversity indices indicated that the populations of *S*. *hirtipupa* have had distinct demographic histories underlying the haplogroups observed in this study. The haplotype diversity was high and nucleotide diversity low for most groups. This suggests that *S*. *hirtipupa* has undergone a rapid population expansion from an ancestral population from its putative center of origin in the northern region of the state of Minas Gerais. This also suggests that the time elapsed has been sufficient for the recovery of haplotype variation via mutation, albeit not long enough for the accumulation of large sequence differences. These indices were low for groups Gr-Goiás and Gr-Central-East_1, indicating that they may have experienced either a prolonged/severe population bottleneck in recent times or selective sweeps [49]. The genetic diversity indices obtained in this study are in line with previous studies on other *Simulium* species [10,19,40], however, no study had been carried out on species from the Neotropical region, where geography and environmental conditions are different from those in the aforementioned studies.

The population expansion is also observed in the mismatch distribution analysis, which shows concordance with the spatial expansion model, since its distribution was unimodal for all groups, except for the group Gr-Central-East_1 that was multimodal. The unimodal distribution showed few pairwise differences in the mismatch distribution analysis, except for group Gr-Bahia for which the pairwise differences observed ranged from 8 to 12 (S3 Fig), indicating that its demographic expansion took place in an earlier period. Also, the negative and significant values of the neutrality test (Fu’s *Fs*) in our study corroborate a recent dispersal of populations of *S*. *hirtipupa* as has been observed for other species [10, 50].

The bimodality or multimodality observed in group Gr-Central-East_1 could indicate that the population has experienced either chronologically distinct expansions or demographic equilibrium [10,51,52]. Bimodality or multimodality may also reflect differences among conspecific individuals from distinct geographic regions [49].

Within group Gr-Goiás, the most frequent haplotype is most likely the ancestral haplotype as it showed the highest number of connections with other haplotypes, which can indicate a recent expansion of those populations [53], which is in line with the genetic diversity results obtained herein. The low genetic diversity indices and the shape of the haplotype network show that the populations within group Gr-Goiás are homogeneous and may have experienced severe bottlenecks as reported for tephritids [54] and culicine mosquitoes [55]. Moreover, alternative explanations may include ancestral contact without sufficient time for divergence to occur, as the average genetic distance was 0.3%. Also, the *Fst* index showed that the populations in the Gr-Goiás group are not genetically similar thus indicating the lack of present gene flow.

The spatiotemporal reconstruction showed the ancestral center of origin of *S*. *hirtipupa* in the northern region of the state of Minas Gerais, followed by dispersal to other regions within the state of Minas Gerais and to the states of Bahia, Pernambuco, and Mato Grosso do Sul around 1.25 Mya. The populations of these states formed distinct groups (Gr-Bahia, Gr-Pernambuco, Gr-Mato Grosso do Sul) that still show low genetic differentiation (average 2.97%) between them when compared to other groups, even though they correspond to a more ancestral dispersal. *S*. *hirtipupa* then dispersed along the eastern coast of Brazil and to the state of Goiás, in the central region of Brazil. From there, *S*. *hirtipupa* dispersed to the state of Tocantins around 874 Kya. Then in a more recent period, dispersal occurred in the opposite direction from the state of Goiás to the state of Minas Gerais and from the eastern Brazilian coast (populations from the states of Espírito Santo and São Paulo) to the states of Minas Gerais and Bahia, and another route to the state of Pernambuco, reaching its present distribution (Fig 7). The routes of dispersal are congruent with the analysis of populational structure, which shows that some groups (Gr-Goiás, Gr-Central-East_1, Gr-Central-East_2 and Gr-Bahia) comprise specimens from different populations indicating that gene flow has occurred at some point in time and space and that these populations are still under a differentiation process.

Our results provide little evidence in support of the hypothesis that rivers acted as barriers in the recent dispersal of *S*. *hirtipupa* in Brazil. The dispersal occurred from the state of Goiás to the state of Minas Gerais and in the opposite route, which could have happened by circumventing the Serra Geral de Goiás and crossing the São Francisco river. Haplotype sharing has been found among these populations, indicating the occurrence of recent gene flow.

In this region, there are reports of other groups that dispersed circumventing the São Francisco river. The species *Lutzomyia longipalpis s*.*l*. (Diptera: Psychodidae) dispersed along the São Francisco river basin from the river’s east bank circumventing its headwaters and going along its west margin and then reaching the costal areas of the Northeast region. This dispersal would have resulted in adaptation to local habitats and population differentiation before the river changed its course [56]. The same kind of dispersal has also been reported for the rodent *Calomys expulsus* (Cricetidae, Sigmodontinae) from the river’s west bank circumventing its headwaters and then reaching the east bank [57].

There are two dispersal routes towards the state of Pernambuco, north of the São Francisco river. The first dispersal route originates from the state of Goiás, and the second originates from the center of origin in the northern part of the state of Minas Gerais. One haplotype is shared between the populations from the states of Pernambuco and Goiás, which can be related either to an ancestral contact or to independent diversification. The high genetic diversity and geographic isolation of this group is concordant with the Carnaval-Moritz model of Pleistocene refugia for the Atlantic Forest [51].

The average genetic distance of the population from Pernambuco when compared to the other populations, excluding the populations from group Gr-Goiás, was lower (3.3%), even when compared to the group Gr-Mato Grosso do Sul (average 2.95%). The population from the state of Pernambuco and the populations from the state of Mato Grosso do Sul are located on opposite ends of the sampled area. Thus, the change in the river course most likely did not have an impact on the dispersal of *S*. *hirtipupa*. Until the Mindel glaciation period (0.4 Mya) the São Francisco river flowed northwards towards the state of Piauí, after which its course changed in the direction of the eastern coast of the Northeast region. After a period of high humidity, it changed its course from endorheic to its present exhoreic pattern [56,58].

Neither the São Francisco river nor the landscape of the state of Bahia acted as geographical barriers for the three populations in the group Gr-Bahia. Population 26BA from the Correntina river, a tributary on the west bank of the São Francisco river, clustered together with specimens from populations 1BA and 15BA, which are east of this river in the Eastern Atlantic Basin of Brazil. Dispersal most likely occurred from population 26BA to 1BA and from this to 15BA. Gene flow was observed in both directions between populations 1BA and 15BA (Fig 7), however, no haplotype sharing was found. The first population (26BA) is located in the Chapadão Ocidental of the São Francisco river, the second population (15BA) in the Chapada Diamantina in an affluent of the Paraguaçu river, and the third (1BA) population on the pre-Litorâneo plateau in Southern Bahia. The state of Bahia has a complex topography as the São Francisco river and large depressions, Depressões Periféricas and Interplanálticas, cut across the Chapadão Ocidental and the Chapada Diamantina, and they are all crisscrossed by the Serra Geral do Espinhaço [59].

Contrary to that which has been reported for other groups of animals, the rivers from the Brazilian east coast have not acted as physical barriers for *S*. *hirtipupa*. The populations of the Atlantic Forest included in this study showed no concordance with a latitudinal division of this biome, except for group Gr-Pernambuco in the northernmost part. These populations clustered into two groups, Gr-Central-East_1 and Gr-Central-East_2, which also encompassed populations from the biomes Cerrado and Caatinga with high genetic similarity (1.37%), indicating the occurrence of gene flow or shared recent history [60]. In the tree, these groups were recovered as sister clades with high support values. As well, their diversification most likely occurred in distinct periods of time, albeit temporally close, the former 1.05 Mya and the latter 0.959 Mya.

Similarly, a study on *Melipona quadrifasciata* (Hymenoptera, Apidae) concluded that, even though its time of divergence was more recent, the river basins on the east coast of the Atlantic Forest also did not act as barriers to gene flow for those populations [50]. On the other hand, the study on the complex *Gymnodactylus darwinii* (Gekkonidae, Squamata) revealed that the rivers Doce, São Francisco, and Paraguaçu (Bahia de Todos os Santos) acted as barriers to gene flow for lizard populations within that complex [61]. Also, modelling studies based on the distribution of vegetation and several groups of animals corroborate the first two rivers as barriers [51]. The São Francisco river also acted as a divide between two groups of populations of the stingless bee *Partamona rustica* (Hymenoptera, Apidae) whose origin was probably on the west bank [62]. Moreover, some groups of small mammals also showed a north-south division along the Atlantic Forest [63]. Contrastingly, regarding the distribution of anurans, it seems that vegetation and climatic conditions have played an important role for the partitioning of this area into ecoregions [64], which are concordant with the terrestrial ecoregions [65].

The populations analyzed in this study partially occupy the terrestrial ecoregions called North and Southeast by Vasconcelos *et al*. (2014) [64]. The population groups also are in agreement with the freshwater ecoregions in Brazil, which are based on the distribution of freshwater fish species [66]. The only exceptions are the populations from western Bahia, from the state of Minas Gerais, and from the eastern coast of the Atlantic Forest that occupied two ecoregions. A previous study on populations from similar regions indicated that the distributions of the cytoforms of *S*. *guianense* are also congruent with the freshwater ecoregions [28].

A probable barrier between groups Gr-Goiás and Gr-Bahia is the Serra Geral de Goiás. The high genetic distance between these groups and the dispersal analysis indicated no gene flow between these populations. The Serra Geral de Goiás, dating back to the Cretaceous [67], and therefore before the diversification of *S*. *hirtipupa*, is the geographic divide between the São Francisco and the Tocantins basins. This watershed divide is a sedimentary plateau that comprises two subunits: the Chapadão Ocidental do São Francisco and Patamares do Chapadão in the state of Goiás [68], which are separated by the Vão do Paranã, a depression between the Serra Geral de Goiás and Planalto do Alto Tocantins-Paranaíba [69,70]. This region has also been identified as a geographical barrier for other groups such as *Pyrrhura pfrimeri* (Aves: Psittacidae) [70] and *Gracilinanus agilis* (Mouse Opossum: Didelphidae) [71].

Some studies point out that elevation may act as a barrier to gene flow [10,72–74]. Elevational barrier was not verified in our study. For example, the three populations in the state of Bahia are clustered together in the group Gr-Bahia and are also present in elevations that range from 179 a 902m (Table 1). Yet, the populations in the state of Minas Gerais present in a lower range of elevations (from 511 to 953m) showed isolation of population 22MG.

Another limitation to dispersal is the flight range of simuliids. Studies point out that the maximum flight range was 400 km and winds can help in their aerial dispersal [75]. Our results reveal that the dispersal distances among populations in this study are much higher than 400 km. The average geographic distance between populations in the groups Gr-Central-East_1 and Gr-Central-East_2 is 515 km, ranging from 136 to 963km. The average genetic distance among these populations is 1.8%, reinforcing the occurrence of recent gene flow with the possible influence of winds in this region.

Climate oscillations in the Pleistocene as well as vegetation expansion and retraction during that period [76] seem to have influenced dispersal. During more humid intervals, more humid environments may have favored dispersal into regions that are currently more arid, such as the Caatinga in the Brazilian Northeast [77].

Our results revealed that, despite the genetic divergence from the Early Pleistocene, not enough time has elapsed for haplotype differentiation, especially on the extremes of the sampled area. Despite this, the data already indicate the presence of cryptic species and that the genetic diversity of some of the groups in the central area are partially associated with geography. Additional studies including populations from unsampled areas as well as nuclear markers will help further elucidate the evolution of *Simulium hirtipupa*.

## Supporting information

S1 Fig Estimate of genetic distance of *Simulium hirtipupa*. Intrapopulation (diagonal and bold) and interpopulation (below the diagonal) genetic distances (%) based on sequencing of a fragment of the mitochondrial COI gene. Analyses were conducted using the K_2_P model.
(PDF)

S2 Fig Pairwise genetic differentiation (*Fst*) between populations of *Simulium hirtipupa*. (PDF)

S3 Fig Graphs of the mismatch distribution to each populational group of *Simulium hirtipupa*. The mismatch distribution for the groups was consistent with the model of demographic expansion, except for the Gr-Central-East_1 group. The lines represent the observed (black line) and expected (grey line) frequency of pairwise differences under the sudden population expansion model.
(PDF)

S1 Table GenBank accession numbers for populations of *Simulium hirtipupa*. Underlined numbers indicate shared haplotypes and in bold haplotypes repeated in the same population.
(PDF)
