# Supplementary material for: Phylogeography and population diversity of Simulium hirtipupa Lutz (Diptera: Simuliidae) based on mitochondrial COI sequences
Source: PLoS One. 2017 Dec 27;12(12):e0190091. doi: 10.1371/journal.pone.0190091 (PMC5744943; doi:10.1371/journal.pone.0190091)
Supplement: S1 Fig — Intrapopulation (diagonal and bold) and interpopulation (below the diagonal) genetic distances (%) based on sequencing of a fragment of the mitochondrial COI gene. Analyses were conducted using the K2P model. (PDF) [file pone.0190091.s001.pdf]

Phylogeography and population diversity of *Simulium hirtipupa* Lutz (Diptera: Simuliidae) based on mitochondrial COI sequences

V. Andrade-Souza†, J. G. Silva†, N. Hamada†

†Instituto Nacional de Pesquisas da Amazônia (INPA), Coordenação de Biodiversidade - CoBio, Laboratório de Citotaxonomia e Insetos Aquáticos, Av. André Araújo, 2936. Petrópolis, Manaus, AM, Brazil, CEP 69067-375. †Universidade Estadual de Santa Cruz, Departamento de Ciências Biológicas, Rodovia Jorge Amado, km 16, Salobrinho, Ilhéus, BA, Brasil, CEP 45662-900.

	1	2	3	4	5	6	7	8	9	10	11	12	13	14	15	16	17	18	19
1. Bahia (01BA)	**2.29**																		
2. Bahia (15BA)	2.39	**1.28**																	
3. Bahia (26BA)	2.57	3.78	**1.50**																
4. Espírito Santo (12ES)	2.44	1.12	3.79	**0.73**															
5. Espírito Santo (15ES)	2.47	1.09	3.90	0.73	**0.71**														
6. Goiás (06GO)	4.14	4.08	4.53	4.20	4.23	**0.16**													
7. Goiás (10GO)	4.02	3.92	4.43	4.03	4.05	0.20	**0.16**												
8. Mato Grosso do Sul (03MS)	3.75	4.02	3.03	4.18	4.12	4.02	4.08	**0.67**											
9. Mato Grosso do Sul (05MS)	3.64	3.89	2.93	4.05	4.02	3.94	4.00	0.64	**0.56**										
10. Mato Grosso do Sul (08MS)	3.69	3.88	3.03	4.03	3.98	3.91	3.98	0.59	0.57	**0.53**									
11. Mato Grosso do Sul (10MS)	3.72	3.90	3.03	4.07	4.02	3.95	4.01	0.61	0.54	0.54	**0.53**								
12. Mato Grosso do Sul (11MS)	3.63	3.86	2.95	4.02	3.96	3.80	3.87	0.59	0.53	0.53	0.51	**0.49**							
13. Minas Gerais (12MG)	2.46	1.09	3.94	0.75	0.66	4.23	4.06	4.22	4.11	4.07	4.10	4.05	**0.65**						
14. Minas Gerais (03MGa)	3.12	2.35	4.07	2.17	2.14	2.56	2.42	4.11	4.01	3.98	4.01	3.93	2.14	**2.76**					
15. Minas Gerais (03MG)	2.49	1.18	3.82	0.75	0.76	4.22	4.04	4.12	3.99	3.97	4.01	3.96	0.78	2.17	**0.83**				
16. Minas Gerais (22MG)	4.30	4.33	4.38	4.06	4.25	4.07	3.99	4.43	4.30	4.30	4.22	4.12	4.29	4.06	4.11	**0.63**			
17. Pernambuco (17PE)	3.14	3.53	2.89	3.52	3.59	4.36	4.23	3.06	2.93	2.97	2.97	2.97	3.62	3.80	3.54	3.68	**0.80**		
18. São Paulo (18SP)	2.56	1.35	3.72	0.86	0.97	4.32	4.15	4.03	3.87	3.88	3.91	3.88	1.03	2.32	0.89	4.03	3.48	**0.92**	
19. Tocantins (28TO)	4.27	4.21	4.59	4.34	4.36	0.35	0.45	4.04	3.95	3.93	3.96	3.83	4.37	2.74	4.34	4.37	4.54	4.43	**0.46**

S1 Fig. Estimate of genetic distance of *Simulium hirtipupa*. Intrapopulation (diagonal and bold) and interpopulation (below the diagonal) genetic distances (%) based on sequencing of a fragment of the mitochondrial COI gene. Analyses were conducted using the K_2P model.
