# Supplementary material for: Phylogeography and population diversity of Simulium hirtipupa Lutz (Diptera: Simuliidae) based on mitochondrial COI sequences
Source: PLoS One. 2017 Dec 27;12(12):e0190091. doi: 10.1371/journal.pone.0190091 (PMC5744943; doi:10.1371/journal.pone.0190091)
Supplement: S2 Fig — (PDF) [file pone.0190091.s002.pdf]

Phylogeography and population diversity of *Simulium hirtipupa* Lutz (Diptera: Simuliidae) based on mitochondrial COI sequences

V. Andrade-Souza†, J. G. Silva†, N. Hamada†

†Instituto Nacional de Pesquisas da Amazônia (INPA), Coordenação de Biodiversidade - CoBio, Laboratório de Citotaxonomia e Insetos Aquáticos, Av. André Araújo, 2936. Petrópolis, Manaus, AM, Brazil, CEP 69067-375. †Universidade Estadual de Santa Cruz, Departamento de Ciências Biológicas, Rodovia Jorge Amado, km 16, Salobrinho, Ilhéus, BA, Brasil, CEP 45662-900.

	1	2	3	4	5	6	7	8	9	10	11	12	13	14	15	16	17	18
1. Bahia (01BA)																		
2. Bahia (15BA)	0.28																	
3. Bahia (26BA)	0.29	0.63																
4. Espírito Santo (12ES)	0.43	0.09	0.70															
5. Espírito Santo (15ES)	0.44	**0.08**	0.71	**0.02**														
6. Goiás (06GO)	0.83	0.86	0.86	0.92	0.92													
7. Goiás (10GO)	0.77	0.82	0.81	0.89	0.90	0.21												
8. Mato Grosso do Sul (03MS)	0.65	0.75	0.64	0.83	0.83	0.92	0.90											
9. Mato Grosso do Sul (05MS)	0.65	0.75	0.64	0.84	0.84	0.93	0.92	**0.04**										
10. Mato Grosso do Sul (10MS)	0.67	0.76	0.66	0.85	0.85	0.93	0.92	**0.01**	**-0.01**									
11. Mato Grosso do Sul (11MS)	0.68	0.77	0.66	0.85	0.85	0.93	0.92	**0.02**	**0.01**	**-0.01**								
12. Mato Grosso do Sul (08MS)	0.67	0.76	0.66	0.84	0.85	0.93	0.92	**-0.02**	**0.04**	**0.02**	**0.03**							
13. Minas Gerais (03MG)	0.43	0.11	0.70	**-0.05**	**-0.01**	0.90	0.88	0.82	0.82	0.83	0.83	0.83						
14. Minas Gerais (22MG)	0.71	0.78	0.76	0.83	0.84	0.92	0.90	0.85	0.86	0.86	0.86	0.86	0.82					
15. Minas Gerais (12MG)	0.45	0.11	0.72	**0.07**	**-0.03**	0.92	0.90	0.84	0.85	0.86	0.86	0.86	**0.05**	0.85				
16. Minas Gerais (03MGa)	**0.20**	0.21	0.53	0.31	**0.31**	0.72	0.61	0.69	0.68	0.70	0.71	0.70	0.29	0.69	0.33			
17. Pernambuco (17PE)	0.61	0.72	0.63	0.78	0.79	0.89	0.86	0.75	0.75	0.76	0.77	0.76	0.77	0.80	0.79	0.68		
18. São Paulo (18SP)	0.42	0.18	0.68	**0.03**	0.16	0.90	0.87	0.80	0.80	0.81	0.82	0.81	**0.02**	0.81	0.24	0.31	0.76	
19. Tocantins (28TO)	0.73	0.79	0.79	0.86	0.87	0.12	0.31	0.86	0.87	0.87	0.88	0.87	0.85	0.87	0.87	0.57	0.85	0.84

S2 Fig. Pairwise genetic differentiation (*Fst*) between the populations of *Simulium hirtipupa*.
