# Supplementary material for: Phylogeography and population diversity of Simulium hirtipupa Lutz (Diptera: Simuliidae) based on mitochondrial COI sequences
Source: PLoS One. 2017 Dec 27;12(12):e0190091. doi: 10.1371/journal.pone.0190091 (PMC5744943; doi:10.1371/journal.pone.0190091)
Supplement: S3 Fig — The mismatch distribution for the groups was consistent with the model of demographic expansion, except for the Gr-Central-East_1 group. The lines represent the observed (black line) and expected (grey line) frequency of pairwise differences under the sudden population expansion model. (PDF) [file pone.0190091.s003.pdf]

Phylogeography and population diversity of *Simulium hirtipupa* Lutz (Diptera: Simuliidae) based on mitochondrial COI sequences

V. Andrade-Souza†, J. G. Silva†, N. Hamada†

†Instituto Nacional de Pesquisas da Amazônia (INPA), Coordenação de Biodiversidade - CoBio, Laboratório de Citotaxonomia e Insetos Aquáticos, Av. André Araújo, 2936. Petrópolis, Manaus, AM, Brazil, CEP 69067-375.
†Universidade Estadual de Santa Cruz, Departamento de Ciências Biológicas, Rodovia Jorge Amado, km 16, Salobrinho, Ilhéus, BA, Brasil, CEP 45662-900.

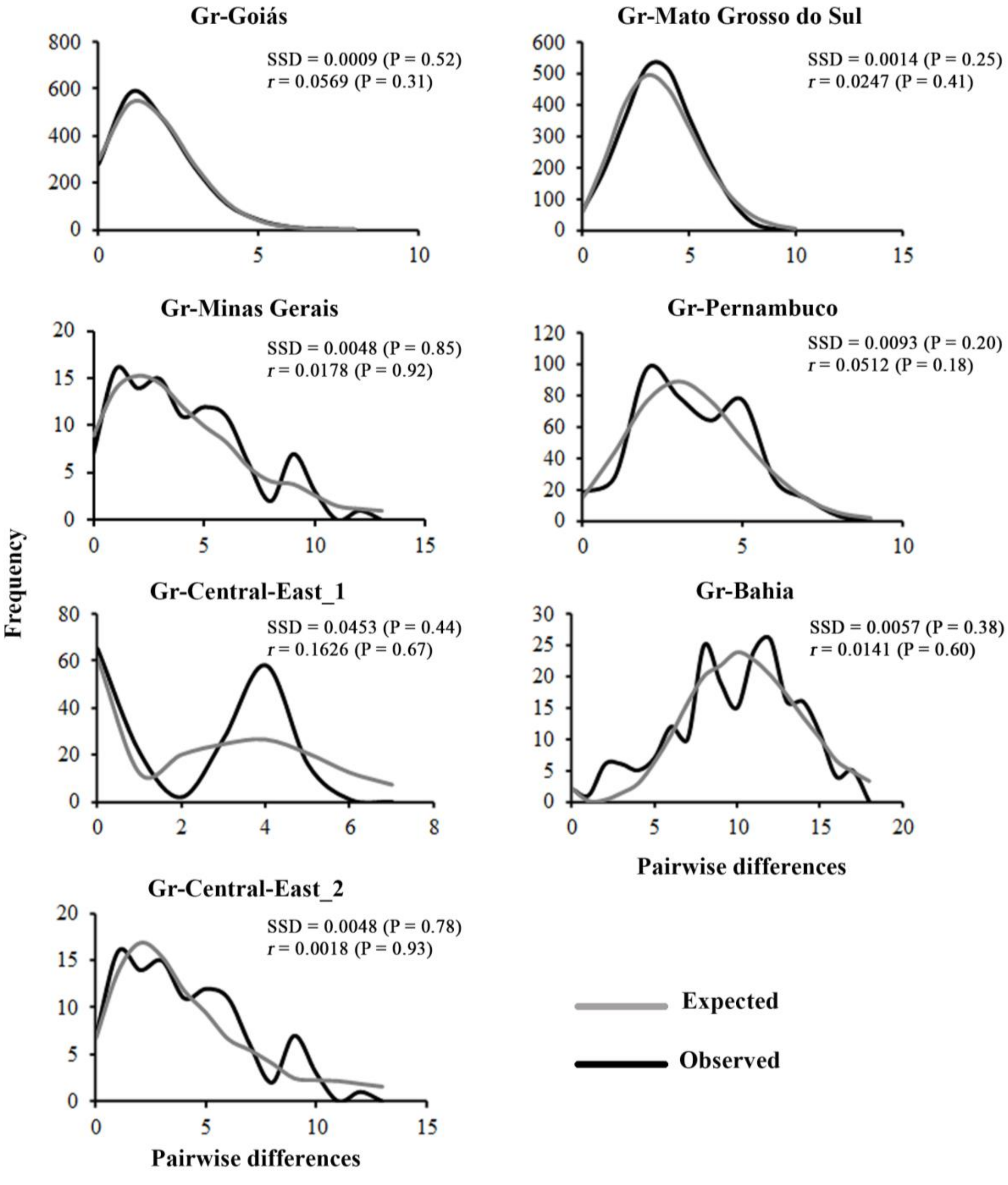

Figure S3 - Graphs of the mismatch distribution to each populational group of *Simulium hirtipupa*. The mismatch distribution for the groups was consistent with the model of demographic expansion, except for the Gr-Central-East_1 group. The lines represent the observed (black line) and expected (grey line) frequency of pairwise differences under the sudden population expansion model.
