# Supplementary material for: Phylogeography and population diversity of Simulium hirtipupa Lutz (Diptera: Simuliidae) based on mitochondrial COI sequences
Source: PLoS One. 2017 Dec 27;12(12):e0190091. doi: 10.1371/journal.pone.0190091 (PMC5744943; doi:10.1371/journal.pone.0190091)
Supplement: S1 Table — Underlined numbers indicate shared haplotypes and in bold haplotypes repeated in the same population. (PDF) [file pone.0190091.s004.pdf]

Table S1 - GenBank accession numbers for populations of *Simulium hirtipupa*. Underlined number indicate shared haplotypes and in bold haplotypes repeated in the same population.

Populations (Code)	GenBank accession numbers
Bahia (01BA)	**MF043593**, MF043594, MF043595, MF043596, MF043597, MF043598, MF043678
Bahia (15BA)	**MF043599**, MF043600, **MF043601**, MF043602, MF043603, MF043604, **MF043605**, MF043606, MF043607
Bahia (26BA)	MF043608, MF043609, MF043610, **MF043611**, MF043612, MF043613, MF043614, MF043615, **MF043616**, MF043617, MF043618, MF043619, MF043620
Espírito Santo (12ES)	MF043621, MF043622, MF043623, MF043626, MF043676, MF043677, MF043678, MF043681, MF043685
Espírito Santo (15ES)	MF043624, MF043625, MF043626, MF043627, MF043628, MF043629, MF043676, MF043677, MF043678, MF043681
Goiás (06GO)	MF043630, MF043631, MF043632, MF043633, MF043634, MF043635, MF043637
Goiás (10GO)	MF043633, MF043635, **MF043636**, MF043637, MF043638
Mato Grosso do Sul (03MS)	MF043639, MF043640, MF043641, MF043642, MF043643, MF043644, MF043645, MF043646, MF043647, MF043648, MF043651, MF043654, MF043665
Mato Grosso do Sul (05MS)	MF043642, MF043649, MF043650, MF043651, MF043652, MF043653, MF043654, MF043655, MF043656, **MF043657**, MF043663
Mato Grosso do Sul (10MS)	MF043641, MF043650, MF043655, MF043662, MF043663, MF043664, MF043665, MF043666, MF043667, MF043668, MF043669, MF043670
Mato Grosso do Sul (11MS)	MF043639, MF043642, MF043650, MF043663, MF043664, MF043666, MF043671, MF043672, MF043673, MF043674, MF043675
Mato Grosso do Sul (08MS)	MF043640, MF043641, MF043644, MF043650, MF043653, MF043658, MF043659, MF043660, MF043661
Minas Gerais (03MG)	MF043676, MF043677, MF043678, MF043679, MF043680, MF043681
Minas Gerais (22MG)	MF043693, MF043694, MF043695, MF043696, MF043697, **MF043698**, MF043699, MF043700, MF043701, **MF043702**, MF043703
Minas Gerais (12MG)	MF043626, MF043677, MF043678, MF043685, MF043686, MF043687, MF043688, MF043689, MF043690, MF043691, MF043692
Minas Gerais (03MGa)	MF043637, MF043682, MF043683, MF043684
Pernambuco (17PE)	MF043635, MF043704, MF043705, **MF043706**, **MF043707**, MF043708, MF043709, MF043710, MF043711, MF043712, MF043713, MF043714, MF043715, MF043716, MF043717, MF043718, MF043719, MF043720, MF043721, MF043722, MF043723, MF043724, MF043725
São Paulo (18SP)	MF043676, MF043677, MF043678, MF043679, MF043681, MF043726
Tocantins (28TO)	MF043727, **MF043728**, MF043729, MF043730, MF043731, MF043732, MF043733, MF043734, MF043735, MF043736, MF043737, MF043738, MF043739, MF043740
